# Neonatal Physical Growth Predicts Electroencephalography Power in Rural South African Children

**DOI:** 10.3390/brainsci14060552

**Published:** 2024-05-29

**Authors:** Amanda R. Tarullo, Denise Evans, Lezanie Coetzee, Diana C. Lopera-Perez, Shaina P. Brady, Laurel J. Gabard-Durnam, Günther Fink, Davidson H. Hamer, Aisha K. Yousafzai, Peter C. Rockers

**Affiliations:** 1 Department of Psychological and Brain Sciences, College of Arts and Sciences, Boston University, Boston, MA 02215, USA; dlopera@bu.edu (D.C.L.-P.); spbrady@bu.edu (S.P.B.); 2 Health Economics and Epidemiology Research Office, Faculty of Health Sciences, University of the Witwatersrand, Johannesburg 2041, South Africa; devans@heroza.org (D.E.);; 3 Department of Psychology, Northeastern University, Boston, MA 02115, USA; l.gabard-durnam@northeastern.edu; 4 Swiss Tropical and Public Health Institute, 4123 Basel, Switzerland; guenther.fink@swisstph.ch; 5 Department of Global Health, Boston University School of Public Health, Boston, MA 02118, USA; dhamer@bu.edu (D.H.H.); prockers@bu.edu (P.C.R.); 6 Section of Infectious Diseases, Department of Medicine, Boston University School of Medicine, Boston, MA 02118, USA; 7 Department of Global Health and Population, Harvard T.H. Chan School of Public Health, Boston, MA 02120, USA; ayousafzai@hsph.harvard.edu

**Keywords:** EEG, socioeconomic status, growth, head circumference, poverty, infancy, low- and middle-income countries, global south, early adversity

## Abstract

Anthropometric measures at birth, indexing prenatal growth, are associated with later cognitive development. Children in low- and middle-income countries (LMIC) are at elevated risk for impaired prenatal and early postnatal growth and enduring cognitive deficits. However, the associations of neonatal physical growth with neural activity are not well-characterized in LMIC contexts, given the dearth of early childhood neuroimaging research in these settings. The current study examined birth length, weight, and head circumference as predictors of EEG relative power over the first three years of life in rural Limpopo Province, South Africa, controlling for postnatal growth and socioeconomic status (SES). A larger head circumference at birth predicted lower relative gamma power, lower right hemisphere relative beta power, and higher relative alpha and theta power. A greater birth length also predicted lower relative gamma power. There were interactions with timepoints such that the associations of birth head circumference and length with EEG power were most pronounced at the 7-month assessment and were attenuated at the 17- and 36-month assessments. The results identify birth head circumference and length as specific predictors of infant neural activity within an under-resourced context.

## 1. Introduction

Fetal growth restriction and stunting in the early postnatal years place children at risk for impaired cognitive development [1,2]. Impaired fetal growth reflects epigenetic and environmental factors in the prenatal environment, such as chronic micro- and macronutrient deficiencies and maternal stress [3,4,5,6]. Slowed postnatal growth likewise is an indicator of chronic malnutrition [7,8]. In combination, impaired prenatal and postnatal growth contribute to stunting, i.e., low height for age. Globally, stunting is present in at least 165 million children under age 5, and wasting is occurring in at least 52 million children, as indexed by low weight for height [7]. In low- and middle-income countries (LMIC), children face numerous co-occurring early adversities [7], and a staggering one out of three young children fails to reach basic developmental milestones [9]. The processes of growth restriction that manifest as stunting also have profound effects on cognitive development [2,8], contributing to this epidemic of unrealized cognitive potential. However, the mechanistic pathways through which growth restriction impacts neural development in LMIC contexts are not well understood, given the scarcity of early childhood neuroimaging studies in these contexts where children are most at risk for poor neurocognitive outcomes. More work is needed to understand the role of normative variations in growth and of impaired growth for neural activity within LMIC contexts.

The implications of both fetal and postnatal growth for cognitive development are well established. In a large-scale study conducted in eight LMIC countries, growth faltering over the first two postnatal years was correlated with cognitive development, with small head circumference being the growth measure most strongly related to cognitive outcomes [2]. Both postnatal stunting (i.e., height for age) and being underweight have been associated with impaired cognitive, motor, and language development in Bangladeshi children [8,10].

In studies that disentangle the effects of prenatal and postnatal growth, it is evident that prenatal growth makes specific contributions to cognitive outcomes over and above postnatal growth. For example, in a study of Finnish children, birth head circumference, length, and weight were all significant predictors of cognitive outcomes, with slower postnatal growth accounting for additional variance in some cognitive domains [1]. In a large study of pregnant women in six countries, fetal cranial growth, determined through ultrasound, uniquely predicted neurocognitive outcomes at age two years [11]. Barker’s fetal origins hypothesis posits that fetal growth conditions have far-reaching influences on lifelong health and well-being via fetal programming [12]. Thus, growth measures at birth can serve both as proxies for prenatal adversity and as predictors of neurodevelopmental trajectories.

The fetal and postnatal environments have irreparable impacts on brain volume, microstructure, neural connectivity, and neurotransmitter function [4,5,13,14]. Prenatal iron deficiency, for example, is linked to intrauterine growth restriction (IUGR) and low birth weight (LBW) and is associated with widespread and lasting impairments in neuronal growth, differentiation, and myelination [4]. Children who are small for their gestational age have been shown to have neonatal abnormalities in EEG activity, including spectral power, which persist into childhood [15].

EEG is recorded from the scalp, indexing neuronal oscillations. EEG spectral power can be partitioned into frequency bands indexing faster and slower neural oscillations, each of which have distinct functional correlates. In high-income countries (HIC), there are age-related changes in the spectral power distribution such that the proportion of spectral power in high-frequency bands increases and the proportion of power in lower-frequency bands decreases with development [16]. Relative EEG power—the proportion of total spectral power in a given frequency band—can therefore be interpreted as indexing neural maturation. These age-related changes are not universal, however, and even within HIC, socioeconomic risk has been associated with altered developmental trajectories of EEG power in the first two postnatal years [17]. EEG developmental trajectories are less well-characterized in LMIC contexts due to the paucity of EEG research in these settings.

There is emerging evidence linking postnatal growth to EEG power and functional connectivity. EEG gamma power, indexing high-frequency oscillations, has been correlated with head circumference in early infancy [13]. In a study in Bangladesh, the postnatal height-for-age z-score was inversely associated with EEG functional connectivity in the theta and beta bands in toddlers, though no such association was observed in an infant cohort [8]. However, the implications of prenatal growth for EEG activity in LMIC contexts are not well-characterized.

The current study leverages a subsample of a birth cohort of Black children in rural Limpopo Province, South Africa [18]. The aim is to test neonatal length, weight, and head circumference as predictors of spontaneous EEG relative power in 7-, 17-, and 36-month laboratory assessments. We statistically control for concurrent growth measures to disambiguate the contributions of restricted prenatal growth from slowed postnatal growth. Further, we control for general socioeconomic status, indexed via household assets within this under-resourced context, to examine the specific role of physical growth in relation to neural activity within the context of general socioeconomic deprivation.

## 2. Materials and Methods

### 2.1. Participants

The sample for the current analyses was comprised of 258 Black children (137 girls) in the Greater Tzaneen subdistrict, Mopani district, Limpopo Province, South Africa. Participants were enrolled in a cluster-randomized controlled trial soon after birth, which took place in the Greater Tzaneen and Greater Giyani subdistricts [18]. The trial was aimed at improving child development through community health worker (CHW) home visits from birth through 24 months, with the endline conducted at 36 months. Caregivers were at least 18 years of age. The current analyses consist of a subsample who participated in laboratory-based assessments of neural function. Invitations to participate in these laboratory-based assessments were restricted to those who (a) lived in the Greater Tzaneen subdistrict, which was closer to the laboratory location in the town of Tzaneen; (b) were born full term (>37 gestational weeks) with a birthweight > 2500 g; and (c) did not have any diagnosed developmental disorder at the time of the first lab assessment at seven months. Participants from this subsample were included in the current analyses if they had useable EEG data from at least two of the three laboratory assessments. Sample characteristics are shown in Table 1.

### 2.2. Procedures

Participants were enrolled in the cluster-randomized controlled trial soon after birth, and a baseline assessment was conducted. Intervention CHWs delivered home-visit interventions from birth to 24 months using a job aid with information on child developmental milestones, health, and nutrition, and strategies to encourage developmentally appropriate play activities. Control CHWs provided the local standard of care on their home visits. The endline was delayed due to the COVID-19 pandemic and occurred at 36 months. The current analyses leverage growth measures obtained at birth, along with EEG and growth data collected from a subsample of participants at 7 months old, 17 months old, and 36 months old. The intervention group was unrelated to EEG relative power measures but was included as a covariate to ensure that treatment would have no impact on the models. Data were collected in a laboratory that was established in the town of Tzaneen. Assessors were extensively trained to collect the data before each of the three timepoints. To enhance the quality of the EEG data, an electromagnetic meter was used to ensure that the assessment rooms had low electrical noise. Study staff scheduled lab visits with caregivers via phone call. On the day of the appointment, participants were transported to and from the lab in a study vehicle. Most caregivers were receptive to the study and to having their infants participate in the EEG assessments. The average time to complete the full assessment was approximately 45 min.

### 2.3. Measures

#### 2.3.1. Electrophysiological Recording and Analysis

At each of the three lab-based assessments—ages 7 months, 17 months, and 36 months—EEG was recorded using a 32-channel portable Geodesic EEG System 400 (by EGI/Philips Neuro, Eugene, OR). As in many LMIC contexts, there were challenges to EEG data collection, particularly the prevalence of load shedding in South Africa, i.e., rolling blackouts due to electrical power shortages, which sometimes required the rescheduling of participants. Impedances were accepted if lower than 50 KOhm. Data were sampled at 500 Hz and referenced to the vertex (Cz). Data were recorded for six minutes while the child sat on the caregiver’s lap with the lights dimmed in the room. This protocol was adopted because the children were unaccustomed to computer screens and in pilot testing appeared overstimulated and distressed by the rotating screensavers often used in baseline EEG data collection with infants and young children. Data were uploaded from laboratory computers to a secure file-sharing platform on a daily basis and regularly checked for data quality.

The raw files were first filtered using NetStation with a 50 Hz notch filter to remove electrical noise and a 70 Hz low pass filter. The data were exported to MATLAB and preprocessed using the Harvard Automated Processing Pipeline for EEG [19]. Data were segmented into two-second epochs. A wavelet-enhanced independent components analysis approach was used to correct for artifacts while retaining the entire length of the data file. Bad channels were replaced using spherical spline interpolation and re-referenced to Cz. Following independent components analysis, observations were excluded from analysis if they had greater than 15% bad channels, more than 5 interpolated channels, fewer than 40 epochs of usable data out of a possible 360 epochs, or median artifact probability greater than 33% for retained independent components.

EEG spectral power was then computed in MATLAB using the Batch Electroencephalography Automated Processing Platform, which is designed to streamline batch EEG processing and increase the reproducibility of results [20]. Power was partitioned into frequency bands, with gamma (30–48 Hz), beta (13–30 Hz), alpha (6–13 Hz), and theta (4–6 Hz) reported here. The delta (1–4 Hz) band was not examined as the infant EEG literature typically has less focus on this slow wave activity. Power was measured in μV^2^/Hz. For each electrode site, these band-level power values were then divided by the total power from 1 to 48 Hz to yield a measure of relative power, i.e., the proportion of total power in that frequency band. The relative power values for all five regions (frontal; parietal; central; temporal; occipital) of both left and right hemispheres were calculated by averaging the appropriate channel measures for each region at each of the three timepoints.

#### 2.3.2. Neonatal Growth Measures

After delivery, families were provided with a *Road to Health* booklet, a free health information booklet provided for all babies at the time of delivery by the South African Department of Health. At this time, clinical staff filled out three measures of growth at birth, which served as indices of prenatal growth: head circumference at birth (cm), birth length (cm), and birth weight (kg). These measures were later extracted from each participant’s *Road to Health* booklet by research staff.

#### 2.3.3. Covariates

Household asset information was collected during the baseline survey. Caregivers were asked to endorse how many of 20 household assets they owned, such as a radio, gas stove, or cellphone. These data were reduced using principal component analysis, generating a score that was then z-scored [21] to index relative SES, such that a family with the mean household assets for this sample would have a z-score of 0. This measure serves as an appropriate index of socioeconomic status (SES) for this sample. Concurrent anthropometric measures of growth were collected by research staff at each of the three laboratory assessments where EEG was collected, with head circumference (cm), length/height (cm), and weight (kg) at each timepoint.

The intervention group was unrelated to any of the EEG relative power and growth measures but was included as a covariate as a precaution, to ensure that treatment would have no impact on the models.

### 2.4. Data Analysis Plan

Hypotheses were tested using a series of repeated measures analyses of variance (RM-ANOVAs), separately for each frequency band. All analyses were conducted using R V4.3.1. [22,23]. RM-ANOVAs were used due to recordings occurring across three timepoints, as well as across multiple regions and two hemispheres. Data were missing at random. Prior to analysis, multiple imputation was used to address missing data (both neural variables and growth variables) with the *mice* package [24]. For all RM-ANOVAs, the relative power for that frequency band was the dependent repeated measure, and within-subjects factors were timepoint (7 months; 17 months; 36 months), region (frontal; parietal; central; temporal; occipital), and hemisphere (right; left). Greenhouse–Geisser corrections were used when the sphericity assumption was violated.

All neonatal growth measures (head circumference at birth; birth length; birth weight) were entered as between-subjects predictors in the RM-ANOVAs, as well as their interactions with timepoint, region, and hemisphere. Next, covariates were added to the models. The number of household assets (z-score), our measure of SES, was added to the models as a between-subjects covariate in order to explore the role of prenatal growth on later EEG power, above and beyond the effects of global sociodemographic risk. Child sex and intervention groups were also added to the RM-ANOVA models as between-subjects covariates.

The interactions of concurrent growth (head circumference; length/height; and birth weight) and timepoint were also added as covariates to account for growth and potential stunting concurrent to EEG recordings, and to parse out the specific effects of prenatal growth.

As EEG power can be affected by data quality, the interactions with the timepoint of the number of usable channels prior to imputation, the number of usable epochs, and the room in which the EEG was conducted were included as between-subjects covariates as well. Controlling for the room was appropriate due to variations in electrical noise levels across testing rooms.

This approach enabled us to test the main effects and interactions of neonatal growth measures in relation to EEG gamma (30–48 Hz), beta (13–30 Hz), alpha (6–13 Hz), and theta (4–6 Hz) power over and above the effects of global environmental risk (SES), child sex, postnatal growth over the first few years of life, and EEG data quality.

## 3. Results

### 3.1. Preliminary Analyses

Descriptive statistics for neonatal and postnatal growth measures are reported in Table 2, along with the World Health Organization’s (WHO) standardized z-score for the sample mean (https://www.who.int/tools/child-growth-standards, accessed on 29 April 2024). If the sample mean is half a standard deviation below the WHO mean for age and child sex, for instance, the z-score would be −0.50. The table also reports the percentage of children for each age, child sex, and growth measure who have impaired growth, defined as greater than two standard deviations below WHO means. Impaired growth would be labeled as stunting for length/height, and underweight for weight. These comparisons to WHO standards are provided to contextualize growth in the current sample.

Descriptive statistics for EEG variables are reported in Table 3. A series of Pearson correlations were conducted to examine the relationship between growth measures at birth, concurrent growth measures at each EEG assessment time, as well as sex and household assets (SES), as shown in Table 4. Concurrent growth measures at the three assessment points were strongly associated with each other, as well as with sex and SES. SES was not related to measures of neonatal growth. Birth length and birth weight were associated with postnatal growth. Please refer to the Supplementary Materials for further details on the neural data, including the correlations of EEG relative power at each timepoint with growth measures, along with power spectra graphs and topographical maps at each timepoint.

### 3.2. Relative Gamma Power and Neonatal Growth

**Figure 1:**
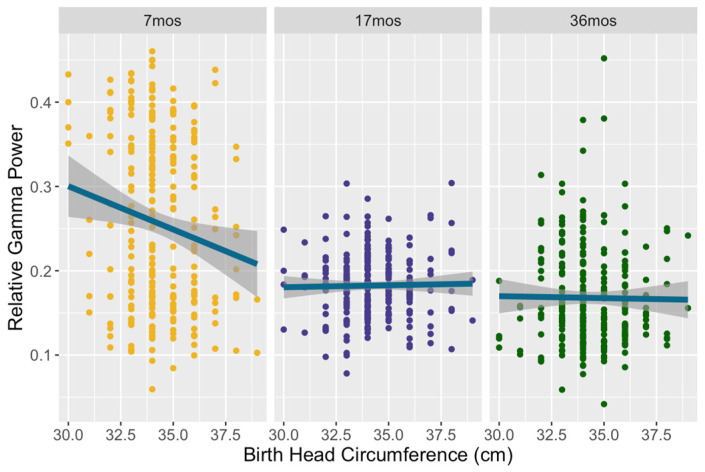
Relative gamma power as a function of birth head circumference across all three timepoints. Children born with a larger head circumference displayed lower relative gamma at seven months.

**Figure 2:**
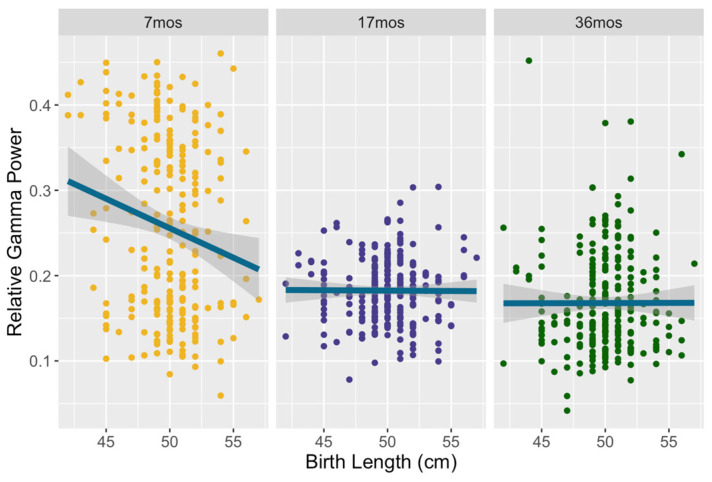
Relative gamma power as a function of birth length across all three timepoints. Children born with a longer birth length displayed lower relative gamma at seven months.

Model 1’s prediction of relative gamma power was significant (F = 34.78, *p* < 0.001) and explained 40.07% of the variance in gamma. There was a main effect of birth head circumference on gamma (30–48 Hz): F(1, 227) = 8.24, *p* = 0.004, partial η^2^ = 0.035. A main effect was also found for birth length: F(1, 227) = 6.91, *p* = 0.009, partial η^2^ = 0.029. Children who were born with a larger head circumference and with greater length demonstrated decreased relative gamma power. For both of these measures of neonatal growth, there was a significant interaction with timepoint: F(2, 484) = 10.85, *p* < 0.001, partial η^2^ = 0.043 and F(2, 484) = 8.8, *p* < 0.001, partial η^2^ = 0.035, respectively. For birth head circumference, the association with gamma power was significant at seven months only, as shown in Figure 1 (F(1, 243) = 25.65, *p* < 0.001, η^2^ = 0.095). The same pattern was seen in birth length, which also was significant only at the 7-month timepoint (F(1, 243) = 21.11, *p* < 0.001, partial η^2^ = 0.08), as shown in Figure 2. No main effect or interactions of birth weight on gamma were found. Concurrent growth measures were unrelated to gamma power in this model, and household assets showed a non-significant trend: F(1, 227) = 3.24, *p* = 0.073, partial η^2^ = 0.014.

### 3.3. Relative Beta Power and Neonatal Growth

Model 2, predicting relative beta power, was significant (F = 8.07, *p* < 0.001) and explained 13.44% of the variance in gamma. There were no main effects of birth head circumference, birth length, or birth weight on beta (13–30 Hz). There was a significant interaction of head circumference at birth with hemisphere: F(1, 254) = 6.35, *p* = 0.01, partial η^2^ = 0.024. The association of birth head circumference with beta power was significant only in the right hemisphere (F(1, 227) = 4.23, *p* = 0.041, η^2^ = 0.018), such that larger head circumference at birth was associated with less relative beta power. This effect did not vary by timepoint. Household assets were not significantly related to beta power in this model, nor were concurrent head circumference or concurrent weight, but concurrent length/height was related (F(1, 237) = 3.67, *p* = 0.013, partial η^2^ = 0.006), such that greater concurrent height was associated with less beta power.

### 3.4. Relative Alpha Power and Neonatal Growth

**Figure 3:**
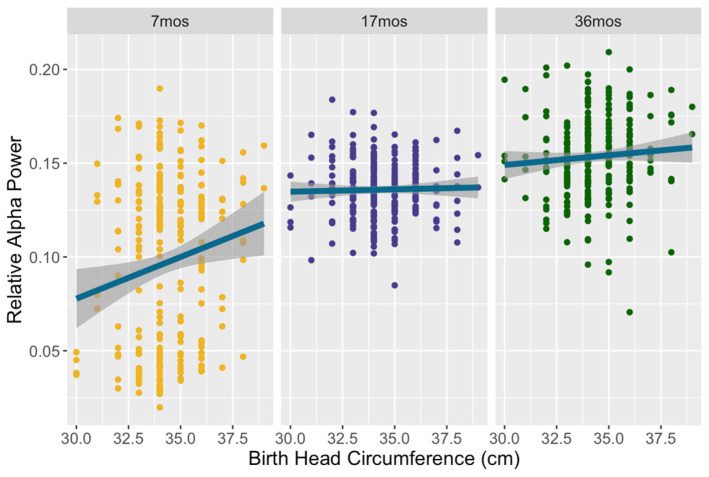
Relative alpha power as a function of birth head circumference across all three timepoints. At 7 months, children born with a larger head circumference displayed higher relative alpha power.

Model 3 examined growth predictors of relative alpha power (F = 45.51, *p* < 0.001) and explained 46.67% of the variance. A main effect was found of birth head circumference on relative alpha power (6–13 Hz): F(1, 227) = 12.96, *p* < 0.001, partial η^2^ = 0.054. This effect was opposite to that of gamma, such that children with a larger head circumference at birth demonstrated higher relative alpha. This effect differed by timepoint (F(2, 484) = 8.05, *p* < 0.001, partial η^2^ = 0.032), such that the relation of birth head circumference and alpha was only significant at the 7-month timepoint, as shown in Figure 3 (F(1, 243) = 18.31, *p* < 0.001, partial η^2^ = 0.07). There were no interactions with hemisphere or region, indicating that the relationship between birth head circumference and gamma was consistent across the brain. Neither birth length nor birthweight was associated with alpha power. Concurrent growth measures were unrelated, but household assets were significantly related to alpha (F(1, 227) = 4.51, *p* = 0.035 partial η^2^ = 0.019), such that children with higher household assets had more relative alpha power.

### 3.5. Relative Theta Power and Neonatal Growth

**Figure 4:**
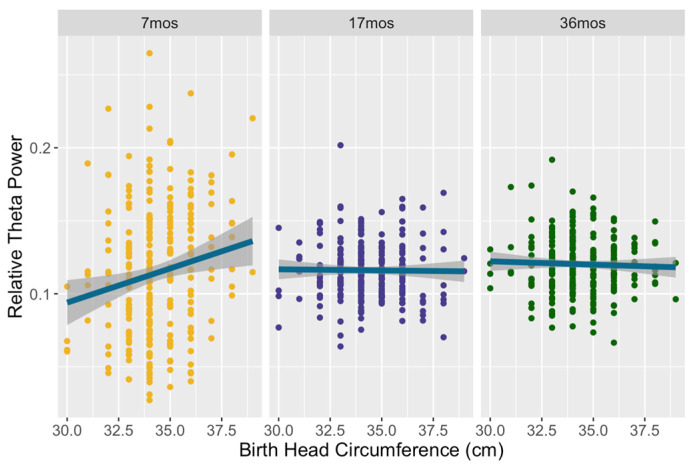
Relative theta power as a function of birth head circumference across all three timepoints. At 7 months, children born with a larger head circumference displayed higher relative theta power.

Model 4 assessed neonatal growth predictors of relative theta power (F = 9.97, *p* < 0.001), with 16.09% of the variance explained. There was a main effect of birth head circumference on relative theta power (4–6 Hz): F(1, 227) = 4.54, *p* = 0.034, partial η^2^ = 0.012. Similar to relative alpha power, children with a larger head circumference at birth demonstrated higher relative theta. This effect also differed by timepoint: F(2, 484) = 9.87, *p* < 0.001, partial η^2^ = 0.039. Similar to the other frequency bands, the relation of birth head circumference to theta was only significant at seven months and not for the latter two timepoints: F(1, 243) = 12.21, *p* < 0.001, partial η^2^ = 0.048. As per Figure 4, children born with a larger head circumference displayed higher relative theta power at seven months. There were no interactions with the hemisphere or region. Concurrent growth measures, as well as household assets, were unrelated to relative theta power.

### 3.6. Follow-Up Analyses

Concurrent head circumference, height, and weight were not associated with EEG relative power in any of the three frequency bands, nor were there any interactions with region and hemisphere. Post-hoc partial correlations were conducted to examine the associations of neonatal and concurrent growth with household assets (SES). When controlling for birth head circumference, concurrent measures of head circumference were significantly related to household assets at seven months (r = 0.15, *p* = 0.018) and 17 months (r = 0.17, *p* = 0.007) but not at 36 months, parsing out the effects of postnatal brain growth. Concurrent height was positively associated with SES at all three timepoints (r = 0.19, *p* = 0.003; r = 0.16, *p* = 0.01; r = 0.19, *p* = 0.002), such that children with more household assets tended to be taller.

## 4. Discussion

The aim of the current study was to investigate the specific contributions of prenatal growth to neural activity within an LMIC context, above and beyond postnatal growth and general SES. We examined birth length, weight, and head circumference as predictors of neural activation at 7, 17, and 36 months among children in rural Limpopo Province, South Africa. While growth measures were within the normal range for the majority of the sample, there was an elevated incidence of impaired growth compared to WHO norms. There was a main effect of birth length on EEG relative gamma power, such that a greater birth length predicted lower relative gamma power. There were main effects of head circumference at birth on EEG relative power, such that a larger head circumference at birth predicted lower relative gamma and higher relative alpha and theta. There was also an interaction with the hemisphere such that a larger head circumference at birth predicted lower relative beta in the right hemisphere. Taken together, the results indicate that children who were longer at birth and had bigger heads, suggesting a more favorable fetal environment, went on to have an EEG pattern characterized by relatively fewer high-frequency oscillations and more slow-wave activity compared to their peers with a smaller birth length and head circumference. Timepoints interacted with the neonatal growth measures in predicting EEG relative power, such that birth length and head circumference were primarily related to 7-month EEG spectral power, and these associations were attenuated at the later assessments. 

The findings are internally consistent, in that larger head circumference at birth predicted relatively fewer high-frequency oscillations (gamma) and more lower-frequency oscillations (alpha and theta). However, these results are the inverse of what one might expect if extrapolating from research in high-income countries, which shows a developmental shift towards more high-frequency activity and less slow-frequency activity [16]. From that perspective, the EEG pattern observed in the infants with better prenatal growth might be interpreted as less mature. It is important to consider the developmental context. In an environment characterized by widespread and persistent material deprivation, it is possible that rapid neural maturation is not advantageous. Rather, extending sensitive periods for brain development could be adaptive. It is plausible that the children who experienced relatively more favorable fetal conditions *within an overall context of significant adversity* were better able to adapt by slowing down postnatal neural maturation. The finding that birth head circumference and birth length were both mainly predictive of 7-month EEG relative power is consistent with this interpretation of a potential maturational lag. Within the 17- and 36-month timepoints, none of the neonatal growth measures predicted EEG activity. To test this speculative interpretation of the data, it would be of interest to extend follow-up into middle childhood to examine both cognitive and EEG trajectories. If the observed EEG pattern of relatively less high-frequency activity at 7 months is indeed adaptive, we would expect it to mediate pathways from neonatal growth to better cognitive outcomes. 

The prior literature has also reported that the interplay of growth and EEG measures can vary across development, including in LMIC contexts. For instance, in Dhaka, Bangladesh, concurrent height-for-age z-score was related to lower EEG theta and beta functional connectivity at 24–36 months of age, but no such association was observed in an infant cohort [8]. The studies differ in LMIC context, type of EEG measure, and whether the focus is on neonatal or concurrent measures of growth. However, taken together, they suggest the need for a nuanced consideration of the interplay of physical growth and neural activity across infancy and early childhood.

A strength of the current study is that concurrent growth, child sex, and general SES, indexed by household assets, were included in the models as covariates. Thus, the results indicate a specific role of neonatal head circumference and length in predicting infant EEG activity. When both neonatal and concurrent growth are included in models, the concurrent growth variables effectively index slower postnatal growth, whereas in studies that do not include birth measurements, the concurrent growth variables are influenced by both prenatal and postnatal growth. In these current analyses, greater concurrent height, indicating faster postnatal growth, was associated with less beta power. There were no other associations of postnatal growth with EEG activity. Post-hoc analyses revealed that while postnatal growth was correlated with household assets in our sample, the neonatal measures of growth were independent of individual differences in household assets within this under-resourced sample. Further research is needed with detailed assessments during the prenatal period to examine what specific environmental factors influence fetal growth conditions and the neonatal growth measures in this context. This sample was enrolled in a cluster-randomized controlled trial aimed at enhancing developmental outcomes. Intervention group status was unrelated to relative power or growth measures and was covaried in the models. Nonetheless, this is a potential limitation.

Head circumference at birth was the most sensitive predictor of EEG relative power, exhibiting associations with all three frequency bands at 7 months. This is consistent with prior research on the importance of fetal cranial growth [11] and is noteworthy given the existing evidence that head circumference is particularly predictive of early cognitive outcomes [2]. Prior research indicates that not only is growth restriction in birth head circumference a risk factor for poorer cognitive outcomes, but also, among children with growth in the normal range, larger birth head circumference positively predicts cognitive achievement [25]. Further, a relatively small proportion of the current sample met the criteria for impaired growth, though the prevalence of impaired growth was elevated compared to WHO norms, particularly for head circumference and length/height. It should be noted that criteria for inclusion in the sample included full-term birth and a birthweight > 2500 g. These initial selection criteria likely reduced the incidence of impaired growth in the current sample, as impaired growth reflects both prenatal growth restriction and postnatal slowing of growth. Given that all participants in the current study were born at full term, smaller neonatal measures were indicative of slower fetal growth, and possibly intrauterine growth restriction in some cases, rather than prematurity. The pattern of associations between birth measures and subsequent neural activity could be different in infants born preterm.

The contributions of measures such as birthweight and postnatal growth could be different within a population experiencing a greater prevalence of stunting and wasting. That said, the finding that birth head circumference predicted relative EEG power even within a full-term sample with a modest incidence of impaired growth suggests that birth head circumference may be a particularly sensitive proxy for the effects of adverse fetal conditions on brain structure and function in LMIC contexts. 

## 5. Conclusions

The current study assessed neural activity in relation to neonatal and postnatal growth in a sample of rural South African infants in a resource-poor context. Within this sample with a modestly elevated risk of impaired growth, a greater head circumference at birth predicted neural activity in infancy, specifically a lower proportion of high-frequency activity and an increased proportion of low-frequency activity. The results indicate the importance of considering both prenatal and postnatal growth indices and exploring the significance of these measures for neural activity within an under-resourced LMIC context.

## Figures and Tables

**Table 1:** Sample demographics.

Variable	M (SD)
Household wealth (z-score)	0.208 (1.012)
Caregiver age at childbirth (years)	31.66 (9.755)
Caregiver education (years)	10.35 (2.42)
Child number of older siblings	1.268 (1.153)

**Table 2:** Growth measures in relation to WHO z-scores by child sex.

**(a) Neonatal**	**Boys**			**Girls**		
	**M (SD)**	**WHO** **z-score**	**% > -2SD WHO**	**M (SD)**	**WHO** **z-score**	**% > -2SD WHO**
Birth Head Circumference	34.31 cm (1.68 cm)	−0.12	4.13%	34.32 cm (1.52 cm)	0.37	2.90%
Birth Length	50.33 cm (2.42 cm)	0.20	6.61%	49.43 cm (2.65 cm)	0.20	10.87%
Birth Weight	3.18 kg (0.39 kg)	−0.35	0%	3.04 kg (0.39 kg)	−0.43	1.45%
**(b) Postnatal**	**Boys**			**Girls**		
	**M (SD)**	**WHO** **z-score**	**% > -2SD WHO**	**M (SD)**	**WHO** **z-score**	**% > -2SD WHO**
7-Month Head Circumference	44.69 cm (1.64 cm)	0.58	4.13%	43.45 cm (1.53 cm)	0.47	2.17%
7-Month Length	69.18 cm (3.13 cm)	0.01	4.96%	67.66 cm (2.66 cm)	0.20	2.17%
7-Month Weight	8.90 kg (1.20 kg)	0.65	3.31%	8.16 kg (1.04 kg)	0.54	2.17%
17-Month Head Circumference	46.67 cm (1.57 cm)	−0.40	9.10%	45.49 cm (1.51 cm)	−0.41	11.59%
17-Month Height	78.07 cm (3.61 cm)	−1.20	28.93%	76.70 cm (2.97 cm)	−1.04	13.77%
17-Month Weight	10.85 kg (1.42 kg)	0.10	5.79%	10.12 kg (1.24 kg)	0.08	1.45%
36-Month Head Circumference	48.60 cm (1.67 cm)	−0.61	6.61%	47.74 cm (1.55 cm)	−0.55	16.67%
36-Month Height	95.21 cm (4.85 cm)	−0.23	8.26%	94.39 cm (3.91 cm)	−0.17	2.17%
36-Month Weight	14.45 kg (1.87 kg)	0.03	0.83%	13.79 kg (1.47 kg)	−0.09	1.45%

**Table 3:** EEG descriptive statistics.

Variable	M (SD)
7mo rel gamma	0.26 (0.11)
7mo rel beta	0.29 (0.05)
7mo rel alpha	0.10 (0.05)
7mo rel theta	0.11 (0.04)
7mo usable 2-s epochs	130.77 (54.94)
17mo rel gamma	0.18 (0.04)
17mo rel beta	0.28 (0.04)
17mo rel alpha	0.14 (0.02)
17mo rel theta	0.12 (0.02)
17mo usable 2-s epochs	106.54 (58.24)
36mo rel gamma	0.17 (0.06)
36mo rel beta	0.27 (0.04)
36mo rel alpha	0.15 (0.02)
36mo rel theta	0.12 (0.02)
36mo usable 2-s epochs	105.74 (59.61)

**Table 4:** Descriptive statistics and correlations for growth and demographic variables.

Variable	*M*	*SD*	1.	2.	3.	4.	5.	6.	7.	8.	9.	10.	11.	12.	13.	14.
1. sex	-	-	-	-	-	-	-	-	-	-	-	-	-	-	-	-
2. household assets	0.21	1.01	−0.10	-	-	-	-	-	-	-	-	-	-	-	-	-
3. birth head circ.	34.31	1.61	−0.00	−0.03	-	-	-	-	-	-	-	-	-	-	-	-
4. birth length	49.93	2.61	−0.18 **	0.10	0.20 **	-	-	-	-	-	-	-	-	-	-	-
5. birth weight	3.12	0.41	−0.14 *	0.06	0.29 **	0.42 **	-	-	-	-	-	-	-	-	-	-
6. 7mo head circ.	44.03	1.70	−0.36 **	0.14 *	0.06	0.18 **	0.15 *		-	-	-	-	-	-	-	-
7. 7mo length	68.38	2.99	−0.25 **	0.20 **	0.05	0.23 **	0.30 **	0.40 **	-	-	-	-	-	-	-	-
8. 7mo weight	8.51	1.18	−0.31 **	0.18 **	0.03	0.21 **	0.25 **	0.62 **	0.59 **	-	-	-	-	-	-	-
9. 17mo head circ.	46.04	1.63	−0.36 **	0.16 **	0.06	0.14 *	0.10	0.63 **	0.27 **	0.46 **	-	-	-	-	-	-
10. 17mo length	77.33	3.35	−0.20 **	0.18 **	0.11	0.27 **	0.27 **	0.34 **	0.59 **	0.53 **	0.34 **	-	-	-	-	-
11. 17mo weight	10.46	1.38	−0.26 **	0.25 **	0.10	0.23 **	0.22 **	0.47 **	0.52 **	0.79 **	0.49 **	0.61 **	-	-	-	-
12. 36mo head circ.	48.14	1.59	−0.28 **	0.10	0.12	0.13 *	0.11	0.61 **	0.29 **	0.43 **	0.65 **	0.31 **	0.39 **	-	-	-
13. 36mo height	94.67	4.47	−0.11	0.21 **	0.02	0.22 **	0.21 **	0.28 **	0.56 **	0.44 **	0.30 **	0.60 **	0.55 **	0.40 **	-	-
14. 36mo weight	14.05	1.66	−0.19 **	0.15 *	0.03	0.25 **	0.23 **	0.44 **	0.48 **	0.65 **	0.48 **	0.48 **	0.74 **	0.57 **	0.73 **	-

* *p* < 0.05; ** *p* < 0.01.

## Data Availability

Data were deposited in the Dryad repository: https://doi.org/10.5061/dryad.qnk98sfm2 (accessed on 13 April 2023).

## Supplementary Materials

The following supporting information can be downloaded at: https://www.mdpi.com/article/10.3390/brainsci14060552/s1, Table S1: Correlations of Relative EEG Power with Growth; Table S2: Absolute Power in ln[μV²/Hz]; Analyses of the associations of growth with absolute EEG power; Figure S1: Average topographic distribution plots across 7, 17, and 36 months for frequency bands Theta (4–6 Hz), Alpha (6–13 Hz), Beta (13–30 Hz) and Gamma (30–50 Hz) after artifact removal; Figure S2: Average power spectra after preprocessing for all electrodes across the scalp at 7, 17, and 36 months. Refs. [26,27,28] are cited in Supplementary Materials.
