# Supplementary material for: Neonatal Physical Growth Predicts Electroencephalography Power in Rural South African Children
Source: Brain Sci. 2024 May 29;14(6):552. doi: 10.3390/brainsci14060552 (PMC11201818; doi:10.3390/brainsci14060552)

Supplemental Materials for: Neonatal Physical Growth Predicts Electroencephalography Power in Rural South African Children

Correlations of EEG relative power and growth measures

Correlations of all growth measures with EEG relative power are reported for the 7-month EEG (Supp. 1a); 17-month EEG (Supp. 1b); and 36-month EEG (Supp. 1c).

Supplemental Table S1. Correlations of Relative EEG Power with Growth

Variable	1.	2.	3.	4.	5.
**1a.**					
1. 7mo rel gamma	-	-	-	-	-
2. 7mo rel beta	.56**	-	-	-	-
3. 7mo rel alpha	-.82**	-.57**	-	-	-
4. 7mo rel theta	-.68**	-.74**	.75**	-	-
5. 7mo useable epochs	.37**	.32**	-.35**	-.32**	-
6. birth head circ.	-.16*	-.10	.16*	.17**	-.02
7. birth length	-.17**	.01	.06	.01	-.02
8. birth weight	-.05	.10	.06	.00	.04
9. 7mo head circ.	-.12	-.16**	.04	.08	-.03
10. 7mo length	-.02	-.11	-.01	.06	-.04
11. 7mo weight	-.03	-.11	.01	.02	-.07
12. 17mo head circ.	-.03	-.18**	.02	.07	.03
13. 17mo length	-.03	-.10	.01	.00	-.00
14. 17mo weight	-.05	-.12	.06	.04	-.05
15. 36mo head circ.	-.08	-.11	.05	.08	.00
16. 36mo height	-.05	-.06	.05	.01	-.05
17. 36mo weight	-.04	-.08	.05	.01	-.03

**1b.**					
1. 17mo rel gamma	-	-	-	-	-
2. 17mo rel beta	.38**	-	-	-	-
3. 17mo rel alpha	-.55**	-.46**	-	-	-
4. 17mo rel theta	-.64**	-.76**	.44**	-	-
5. 17mo useable epochs	.18**	.06	-.09	-.15*	-
6. birth head circ.	.02	.03	.03	-.01	-.10
7. birth length	-.01	.04	.01	-.04	-.05
8. birth weight	-.01	.03	-.04	-.02	.05
9. 7mo head circ.	-.06	-.04	.10	.10	.04
10. 7mo length	-.03	-.07	.04	.03	-.01
11. 7mo weight	-.05	-.07	.11	.09	-.11
12. 17mo head circ.	-.04	.01	.03	.03	.03
13. 17mo length	-.04	-.06	.09	.03	.00
14. 17mo weight	-.01	-.03	.09	.01	-.10
15. 36mo head circ.	-.07	-.03	.08	.09	.01
16. 36mo height	-.02	.02	.03	.02	-.03
17. 36mo weight	-.01	.00	.07	-.01	-.11

**1c.**					
1. 36mo rel gamma	-	-	-	-	
2. 36mo rel beta	.08	-	-	-	
3. 36mo rel alpha	-.38**	-.29**	-	-	
4. 36mo rel theta	-.31**	-.52**	.39**	-	
5. 36mo useable epochs	.13*	-.00	.39**	-.11	
6. birth head circ.	-.01	-.06	.08	-.04	-.03
7. birth length	.00	.07	.14*	.01	-.06
8. birth weight	.00	.02	.10	.01	-.01
9. 7mo head circ.	-.05	.01	.13*	.06	.03
10. 7mo length	-.06	-.05	.09	.09	-.01
11. 7mo weight	-.03	-.05	.13*	.10	.01
12. 17mo head circ.	-.04	.03	.14*	-.01	-.05
13. 17mo length	-.03	-.05	.11	.09	-.02
14. 17mo weight	-.04	-.03	.13*	.08	-.08
15. 36mo head circ.	-.07	.07	.06	.08	.06
16. 36mo height	-.06	.11	.03	.00	-.08
17. 36mo weight	-.06	.06	.10	.03	-.07

***p < .05; **p < .01**

Association of neonatal and postnatal growth measures to EEG absolute power

The main analyses focus on relative EEG power, the proportion of total spectral power in a given frequency band. Absolute power, in contrast, is an index of the total neural activity in a frequency band [26]. In the interest of completeness, we report as supplementary analyses parallel RM-ANOVAs for absolute power, conducted with the same between-subjects and within-subjects factors as the main analyses. When developmental studies report analyses with both absolute and relative power, the pattern of findings often differs across these two distinct measures [26–28])=, and that is the case here as well. Descriptive statistics for absolute power are reported in Supplemental Table 2.

Supplemental Table S2. Absolute Power in ln[μV²/Hz]

**(a) 7 Months**	**M (SD)**
Absolute Gamma Power (30-48 Hz)	-2.89 (1.20)
Absolute Beta Power (13-30 Hz)	-2.64 (1.48)
Absolute Alpha Power (6-13 Hz)	-2.33 (2.08)
Absolute Theta Power (4-6 Hz)	-1.54 (1.90)
**(b) 17 Months**	**M (SD)**
Absolute Gamma Power (30-48 Hz)	-1.48 (0.42)
Absolute Beta Power (13-30 Hz)	-.97 (0.37)
Absolute Alpha Power (6-13 Hz)	- 0.11 (0.34)
Absolute Theta Power (4-6 Hz)	0.29 (0.35)
**(c) 36 Months**	**M (SD)**
Absolute Gamma Power (30-48 Hz)	-1.76 (0.52)
Absolute Beta Power (13-30 Hz)	-1.08 (0.41)
Absolute Alpha Power (6-13 Hz)	- 0.07 (0.43)
Absolute Theta Power (4-6 Hz)	0.25 (0.46)

Absolute Gamma Power and Neonatal Growth

There was a main effect of head circumference on absolute gamma (30-48 Hz), F(1, 227) = 9.86, p = 0.002, $\eta^2 = 0.04$, such that children born with a larger head circumference demonstrated increased absolute gamma power. This effect differed by timepoint, F(2, 484) = 5.21, p = .006, $\eta^2 = 0.02$, only significant at the seven month timepoint, F(1,243) = 12.7, p <.001, $\eta^2 = 0.05$.

Absolute Beta Power and Neonatal Growth

There was a main effect of head circumference on absolute beta (13-30 Hz), F(1, 227) = 14.59, p < 0.001, $\eta^2 = 0.06$, such that children born with a larger head circumference demonstrated increased absolute beta power. This effect differed by timepoint, F(2, 484) = 11.39, p < .001, $\eta^2 = 0.045$. This association was significant at seven months only, F(1, 243) = 18.46, p = <.001, $\eta^2 = 0.07$).

Absolute Alpha Power and Neonatal Growth

There was a main effect of head circumference on absolute alpha (6-13 Hz), F(1, 227) = 24.33, p < 0.001, $\eta^2 = 0.09$, such that children born with a larger head circumference demonstrated increased absolute alpha power. This effect differed by timepoint F(2, 484) = 21.03, p < 0.001, $\eta^2 = 0.08$, with the association

only significant at the seven month timepoint, $F(1, 243) = 29.18$, $p = <.001$, $\eta^2 = 0.11$. The association of alpha power and head circumference also differed by hemisphere $F(1, 254) = 6.25$, $p = .013$, $\eta^2 = 0.02$. While this association was significant in the left hemisphere, $F(1, 227) = 16.9$, $p <.001$, $\eta^2 = 0.07$, there was a larger effect in the right hemisphere $F(1, 227) = 30.21$, $p <.001$, $\eta^2 = 0.12$.

Absolute Theta Power and Neonatal Growth

There was a main effect of head circumference on absolute theta (4-6 Hz), $F(1, 227) = 17.63$, $p <.001$, $\eta^2 = 0.07$, such that children born with a larger head circumference demonstrated increased absolute theta power. This effect differed by timepoint, $F(2, 484) = 16.98$, $p < .001$, $\eta^2 = 0.07$. This association was significant at seven months only, $F(1, 243) = 24.23$, $p <.001$, $\eta^2 = 0.09$. This effect also differed by hemisphere, $F(1, 254) = 5.29$, $p = .02$, $\eta^2 = 0.02$. While it was significant in the left hemisphere, $F(1, 227) = 11.55$, $p < .001$, $\eta^2 = 0.05$, there was a larger effect in the right hemisphere, $F(1, 227) = 22.01$, $p <.001$, $\eta^2 = 0.04$. In addition to head circumference at birth, there was also a main effect of birth weight $F(1, 227) = 4.66$, $p = .03$, $\eta^2 = 0.02$, such that a higher birthweight was associated with less absolute theta. This association also differed by timepoint, $F(2,484) = 3.49$, $p = .03$, $\eta^2 = .014$, only significant at seven months, $F(1,243) = 5.46$, $p = .02$, $\eta^2 = 0.02$. Lastly, there was a interaction of birth length and timepoint, $F(2, 484) = 3.44$, $p = .03$, $\eta^2 = .014$, such that the relation was significant at seven months only, $F(1 ,243) = 4.31$, $p = .034$, $\eta^2 = 0.017$, with higher birth length associated with higher levels of absolute theta power.

Supplemental Figure S1. Average topographic distribution plots across 7, 17, and 36 months for frequency bands Theta (4-6Hz), Alpha (6-13Hz), Beta (13-30Hz) and Gamma (30-50Hz) after artifact removal.

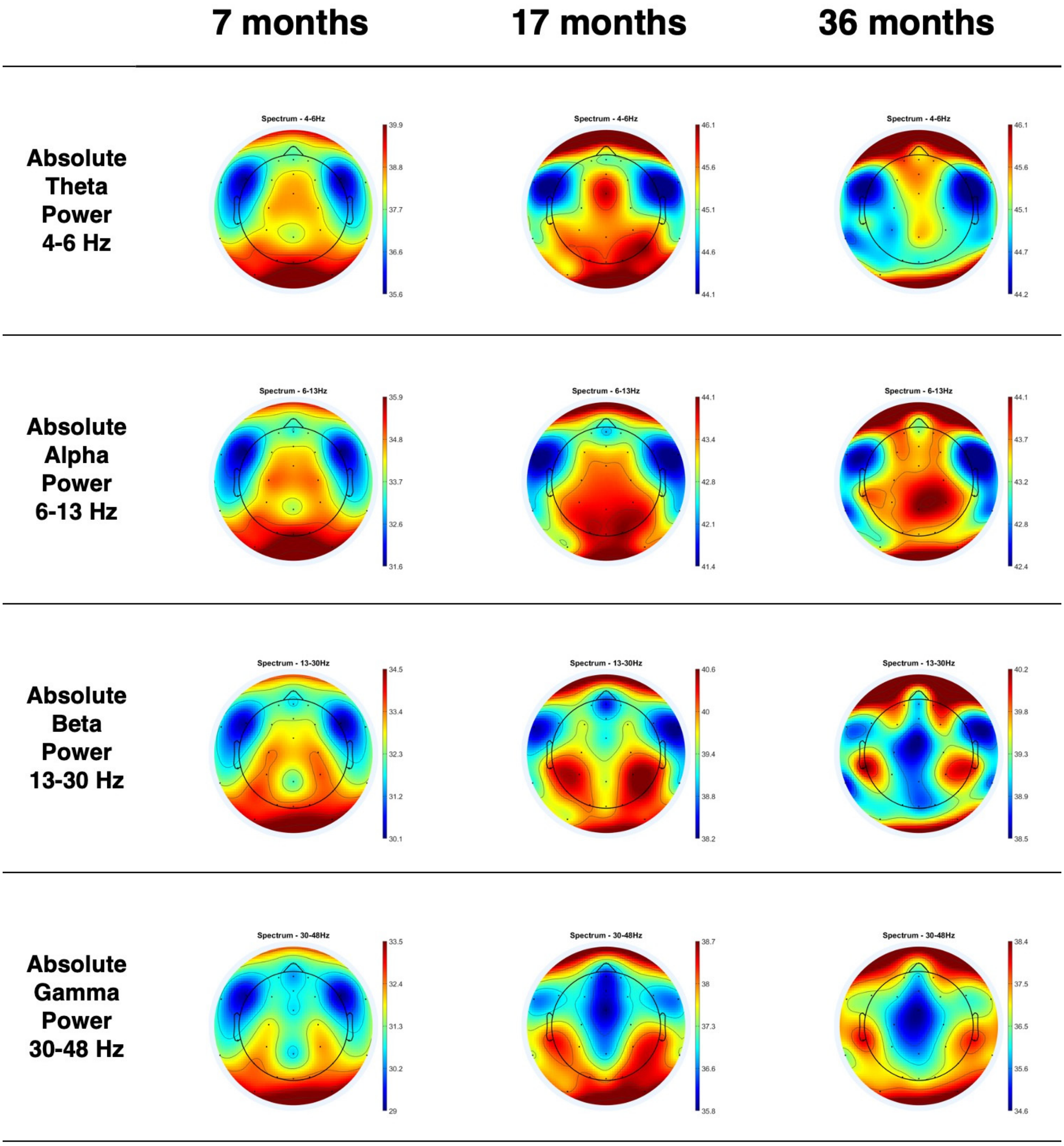

Supplemental Figure S2. Average power spectra after preprocessing for all electrodes across the scalp at 7, 17, and 36 months.

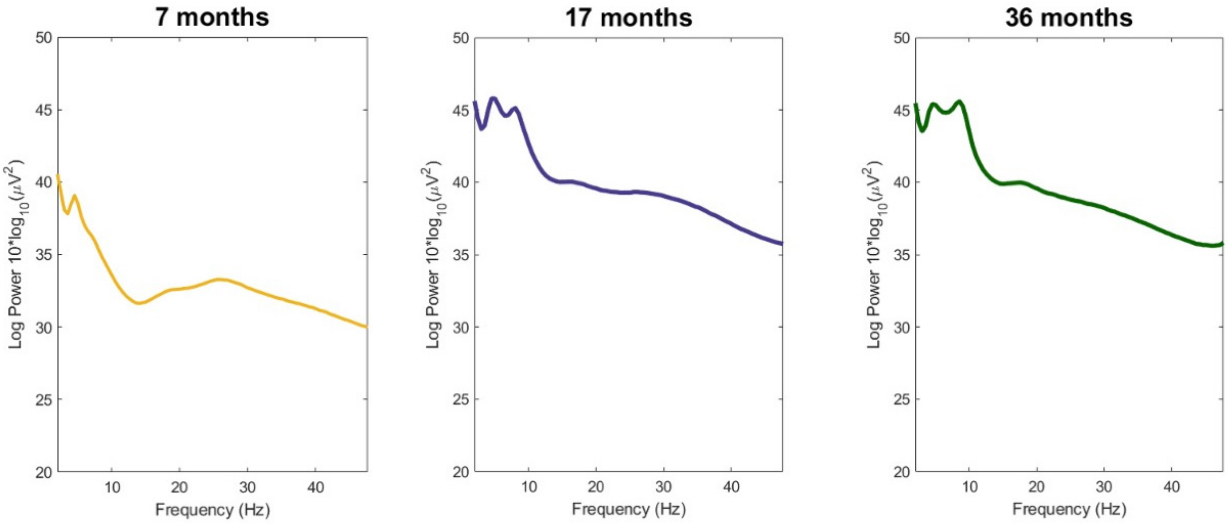

Supplement: Supplementary file 1 [file brainsci-14-00552-s001.zip › brainsci-2963561-supplementary.pdf]
